# Suppression of TGF-β_1_/Smad Signaling Pathway by Sesamin Contributes to the Attenuation of Myocardial Fibrosis in Spontaneously Hypertensive Rats

**DOI:** 10.1371/journal.pone.0121312

**Published:** 2015-03-20

**Authors:** Mengqiu Zhao, Shuguo Zheng, Jieren Yang, Yuanjie Wu, Younan Ren, Xiang Kong, Wei Li, Jiali Xuan

**Affiliations:** 1 Department of Pharmacology, Wannan Medical College, Wuhu, 241002, China; 2 Department of Basic Theory of Chinese Medicine, Anhui University of Chinese Medicine, Hefei, 230038, China; Universidade Federal do Rio de Janeiro, BRAZIL

## Abstract

This study investigated the effect of sesamin on myocardial fibrosis in spontaneously hypertensive rats (SHRs) and the possible mechanisms involved. Twenty-eight male SHRs were randomly allocated to SHR group, Ses160 group (sesamin 160 mg/kg), Ses80 group (sesamin 80 mg/kg) and Cap30 group (captopril 30 mg/kg). Seven male WKY rats were used as control. Sesamin and captopril were administered intragastrically for 12 weeks. Captopril significantly reduced systolic blood pressure and angiotensin II (Ang II) levels in SHRs, accompanied by a marked attenuation of left ventricular hypertrophy (LVH) and collagen deposition (*P* <0.05 or *P* <0.01). Though sesamin had no significant influence on Ang II levels, and the hypotensive effect was also significantly inferior to that of captopril (*P* <0.05 or *P* <0.01), however, the improvement of LVH and collagen deposition was similar to that in captopril group. Sesamin markedly reduced transforming growth factor-β_1_ (TGF-β_1_) content in cardiac tissues, with Smad3 phosphorylation decreased and Smad7 protein expression increased notably (*P* <0.05 or *P* <0.01). Protein expression of type I collagen and type III collagen, target genes of Smad3, was down-regulated markedly by sesamin (*P* <0.05 or *P* <0.01). In addition, sesamin significantly increased total antioxidant capacity and superoxide dismutase protein in cardiac tissues (*P* <0.05 or *P* <0.01), while the expression of NADPH oxidase subunit p47phox and malondialdehyde content were reduced markedly (*P* <0.05 or *P* <0.01). In vitro studies also demonstrated that sesamin was able to suppress Ang II induced phosphorylation of Smad3 and secretion of TGF-β_1_ and type I and type III collagen in cultured rat cardiac fibroblasts. These data suggest that sesamin is capable of attenuating hypertensive myocardial fibrosis through, at least partly, suppression of TGF-β_1_/Smad signaling pathway.

## Introduction

Hypertension, or high blood pressure, can induce serious damage to heart, brain and kidney, with hypertensive heart disease being the leading cause of death from hypertension. Chronic hypertension can cause left ventricular hypertrophy (LVH) through neural and humoral factors besides increased cardiac workload [1]. Increasing evidence indicated that, in addition to compensatory cardiomyocyte hypertrophy, myocardial fibrosis makes considerable contribution to left ventricular hypertrophy and accounts mainly for the development of left ventricular diastolic and systolic dysfunction and ultimate heart failure [2], suggesting that suppression of myocardial fibrosis would be an effective approach to improve hypertensive heart disease.

Previous studies have demonstrated that various factors are involved in the development of hypertensive myocardial fibrosis, with angiotensin II (Ang II) being the most important one [3]. As the effector of renin-angiotensin system (RAS), Ang II can induce fibrosis by promoting extracellular matrix (ECM) deposition [4]. A large amount of evidence indicated that excessive activation of both systemic and local RAS occurs during hypertension, resulting in a significant increase of Ang II in cardiac tissues [5]. Excessive Ang II can stimulate fibroblasts proliferation, differentiation into myofibroblasts and collagen secretion [6]. The findings that angiotensin converting enzyme inhibitors (ACEI) can effectively alleviate myocardial fibrosis in hypertension provide further evidence for the critical role of Ang II in hypertensive myocardial fibrosis.

Recent studies have shown that Ang II induced myocardial fibrosis in hypertension required the participation of transforming growth factor-β_1_ (TGF-β_1_) [7], one of the strongest pro-fibrotic factors that can induce fibroblasts proliferation, differentiation and collagen synthesis [8]. Ang II can induce myocardial fibrosis through up-regulation of TGF-β_1_ expression and subsequent activation of TGF-β_1_/Smad signaling pathway [9]. In addition, Ang II can activate nicotinamide adenine dinucleotide phosphate (NADPH) oxidase, a main source of endogenous reactive oxygen species (ROS), resulting in a significant increase of intracellular ROS [10], which mediates myocardial fibrosis by stimulating TGF-β_1_ secretion and promoting cardiac fibroblasts proliferation and collagen synthesis [11]. The findings that antioxidants can suppress Ang II induced collagen synthesis in cardiac fibroblasts and improve cardiac function in hypertensive rats provide a potential target for the prevention of myocardial fibrosis in hypertension with antioxidants [12,13].

Sesamin (> 98%, Shanghai Pureone Biotechnology, Shanghai, China), a major lignan from sesame seeds, has been shown to possess antioxidant and cardioprotective properties [14,15]. Our previous study demonstrated that sesamin was capable of alleviating left ventricular remodeling in spontaneously hypertensive rats (SHRs) [16], with the precise mechanisms remaining unclear. In the present study, we demonstrated that sesamin can suppress myocardial fibrosis in SHRs through inhibition of TGF-β_1_/Smad signaling pathway, which might be related to its antioxidant capacity.

## Materials and Methods

### Animal grouping and administration

Twenty-eight male SHRs at the age of 12 weeks and 7 male Wistar-Kyoto (WKY) rats of the same age (Slac laboratory Animal Co., Shanghai, China) were maintained in a temperature and humidity controlled room with a 12 h light-dark cycle. Rats were fed a standard diet with free access to drinking water. After adaptive feeding for 1 week, SHRs were randomly allocated to 4 groups, namely SHR group, high dose of sesamin group (Ses160, 160 mg/kg), low dose of sesamin group (Ses80, 80 mg/kg) and captopril group (Cap30, 30 mg/kg), with WKY rats used as a control. Rats in sesamin and captopril groups were administered intragastrically with corresponding drugs suspended in 0.5% sodium carboxymethyl cellulose (CMC-Na) for 12 weeks, while those in WKY and SHR groups were given 0.5% CMC-Na of the same volume. Doses of medication were adjusted according to the changes in body weight monitored weekly. Systolic blood pressure (SBP) was measured biweekly with tail-cuff method (ALC-NIBP, Shanghai, China). This study was conducted in accordance with the Guide for the Care and Use of Laboratory Animals and approved by the Committee on the Care and Use of Laboratory Animals of Wannan Medical College.

### Assessment of left ventricular mass index

At the end of the experiment, rats were anaesthetized with intraperitoneal injection of pentobarbital sodium (40 mg/kg) after overnight fasting. Following blood sample collection from abdominal aorta, rat hearts were removed and rinsed with ice-cold normal saline. After drying with filter paper, left ventricles were dissected and weighted to calculate left ventricular mass index (LVMI), which was defined as the ratio of left ventricular weight to body weight (mg/g).

### Masson staining protocol [17]

Left ventricular tissues of the same region were fixed in 4% paraformaldehyde at room temperature overnight, followed by dehydration with gradient alcohol and embedding in paraffin. Sections of 5 μm were subjected to Masson staining according to the manufacturer’s instructions (Loogene Biotechnology Co., Beijing, China). In Masson-stained sections, myocardial cells were stained red while collagen was stained blue. Six randomly selected microscopic fields of each section were analyzed for collagen deposition using Image-Pro Plus 6.0 (Media Cybernetics MD, USA), which was expressed as collagen volume fraction (CVF), the percentage of the area stained blue for collagen to the total area of each microscopic field. The CVF of each animal represents the mean of 6 randomly selected microscopic fields.

### Plasma Ang II assay

Following collection of blood samples into EDTA coated tubes added with phenylmethanesulfonyl fluoride (PMSF, 1 mmol/L, Sigma-Aldrich, MO, USA), plasma was separated by centrifugation at 3000 rpm for 10 min at 4°C. Levels of Ang II were determined by enzyme-linked immunosorbent assay (ELISA, Biocalvin Company, Suzhou, China) according to the manufacturer’s instructions.

### Myocardial biochemical analysis

Left ventricular tissues were homogenized in 10 mmol/L ice-cold phosphate buffered saline supplemented with 1 mmol/L PMSF, followed by centrifugation at 12000 g for 10 min at 4°C. Levels of total antioxidant capacity (TAC) was determined by ferric reducing/antioxidant power test (FRAP assay) using commercially available kits (Nanjing Jiancheng Bioengineering Institute, Nanjing, China). Each 0.01 increment of the absorbance at 595 nm of reaction system induced by 1 ml homogenate in 1 min was defined as one TAC unit. Malondialdehyde (MDA) contents were assayed by thiobarbituric acid assay (Nanjing Jiancheng Bioengineering Institute, Nanjing, China). Contents of TGF-β_1_ (Lengton Biotechnology, Shanghai, China), Ang II, type I and type III collagen were assayed by ELISA (all from Biocalvin Company, Suzhou, China). All results were normalized to protein content in the supernatants determined by bicinochoninic acid (BCA) assay (Beyotime Biotech, Haimen, China).

### Western blot analysis

Total protein of left ventricular tissue was extracted with RIPA lysis buffer (Beyotime Biotech, Haimen, China) supplemented with 1 mmol/L PMSF, followed by determination of protein concentrations with BCA protein assay kit. Then protein samples were subjected to SDS-polyacrylamide gel electrophoresis (PAGE) and transferred to nitrocellulose membrane (Millipore, MA, USA). After blocking with 5% skimmed milk in Tris buffered saline containing 0.5% Tween-20 (TBST), the membrane was incubated overnight at 4°C with primary antibodies against superoxide dismutase (SOD, 1:1000), Smad3 (1:3000), p-Smad3 (phospho Ser 423/425, 1:2000), Smad7 (1:1000, Abcam, Cambridge, UK) and tubulin (1:1000, Beyotime Biotech, Haimen, China), followed by incubation with horseradish peroxidase (HRP) conjugated secondary antibody (Beyotime Biotech, Haimen, China) for 1 h at room temperature. Bound HRP was visualized on an X-ray film with an enhanced chemiluminescence substrate (ECL, Beyotime Biotech, Haimen, China). Protein bands were analyzed with Quantity One software (Bio-Rad, USA) and the level of each protein was normalized to that of tubulin except for that of p-Smad3, which was normalized to that of Smad3.

### Isolation and culture of rat cardiac fibroblasts

To explore whether the anti-fibrotic effect of sesamin in SHRs was dependent on its blood pressure lowering effect, we carried out in vitro studies on rat cardiac fibroblasts (CFs). CFs were isolated from 0 to 3-day-old neonatal rats as described previously [18]. CFs were cultured in DMEM medium (HyClone, Utah, USA) supplemented with 10% fetal bovine serum (Gibco, MD, USA) in a humidified atmosphere of 95% air and 5% CO_2_ at 37°C. CFs were identified by characteristic morphology and immunochemical staining for vimentin.

### Determination of TGF-β_1_ and collagen contents in culture supernatants

CFs were plated into 24-well plates at a density of 1×10^5^ cells/well and cultured to near confluence. Following incubation with indicated concentrations of sesamin (1.0, 10.0 and 100.0 μmol/L) for 12 h, cells were exposed to Ang II (0.1 μmol/L) for 24 h. Culture supernatants were collected and analyzed for TGF-β_1_ and type I and type III collagen by ELISA according to the manufacturer’s instructions.

### Evaluation of Smad3 phosphorylation

CFs were plated into 6-well plates at a density of 1×10^6^ cells/well and treated as described in 2.8. After washing twice with pre-cooled PBS, cells were lyzed on ice with RIPA lysis buffer (Beyotime Biotech) containing 1 mmol/L PMSF (Sigma). Cell lysates were assayed for protein concentration with BCA protein assay kit, followed by evaluation of Smad3 and p-Smad3 by western blot analysis as detailed in 2.6.

### Statistical analysis

All data were presented as mean ± SD. Statistical analysis were performed by one-way ANOVA followed by Bonferroni’s multiple comparison test. A value of *P* < 0.05 was considered statistically significant. All statistical analysis was performed using SPSS 21.0 statistical software package (Chicago, IL, USA).

## Results

### Effect of sesamin on systolic blood pressure (SBP)

**Fig 1:**
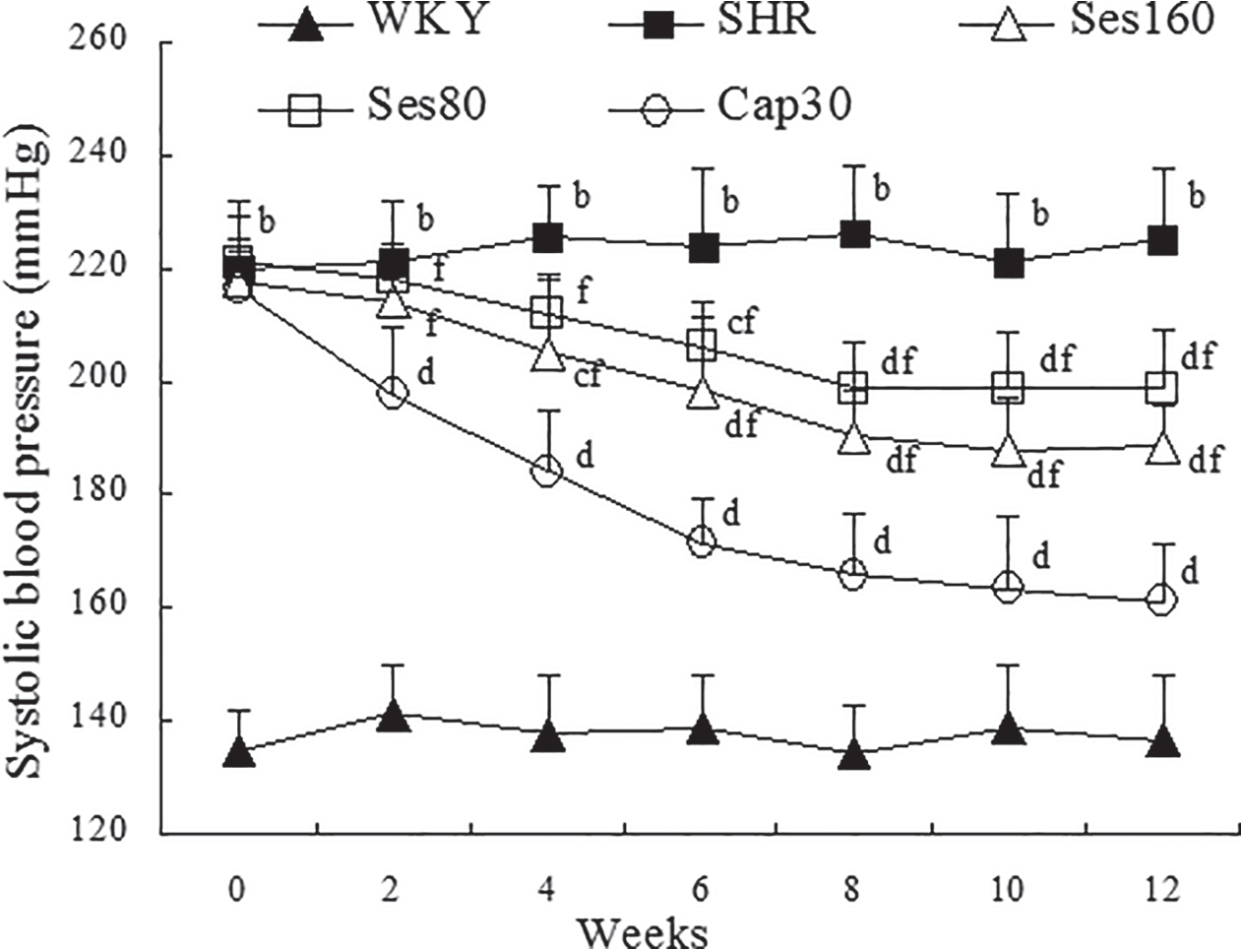
Effect of sesamin on systolic blood pressure (SBP) in rats. Sesamin and captopril were administered to rats intragastrically for 12 weeks, with SBP monitored biweekly by tail-cuff method. Data were presented as mean±SD (n = 7). b *P* <0.01 vs. WKY group; c *P* <0.05, d *P* <0.01 vs. SHR group; f *P* <0.01 vs. Cap30 group.

As shown in Fig. 1, the mean SBP in SHRs remained notably higher than that in WKY rats all through the experiment (*P* <0.01), while in captopril supplemented group, a marked and continuous decrease in SBP was observed since the second week of treatment (*P* <0.01). By contrast, high dose of sesamin exhibited evident hypotensive effect from the fourth week of supplementation (*P* <0.05 or *P* <0.01), whereas low dose of sesamin did not show significant hypotensive effect until the sixth week of treatment (*P* <0.05 or *P* <0.01). Furthermore, SBP in sesamin supplemented groups were significantly higher than that in captopril group during the whole period of treatment (*P* <0.01). Both sesamin and captopril can induce only partial reduction of SBP in SHRs as compared to that in WKY group. These results indicated that, though sesamin exhibits significant hypotensive effect in SHRs, it is notably inferior to captopril in reducing blood pressure.

### Effect of sesamin on left ventricular mass index (LVMI)

**Fig 2:**
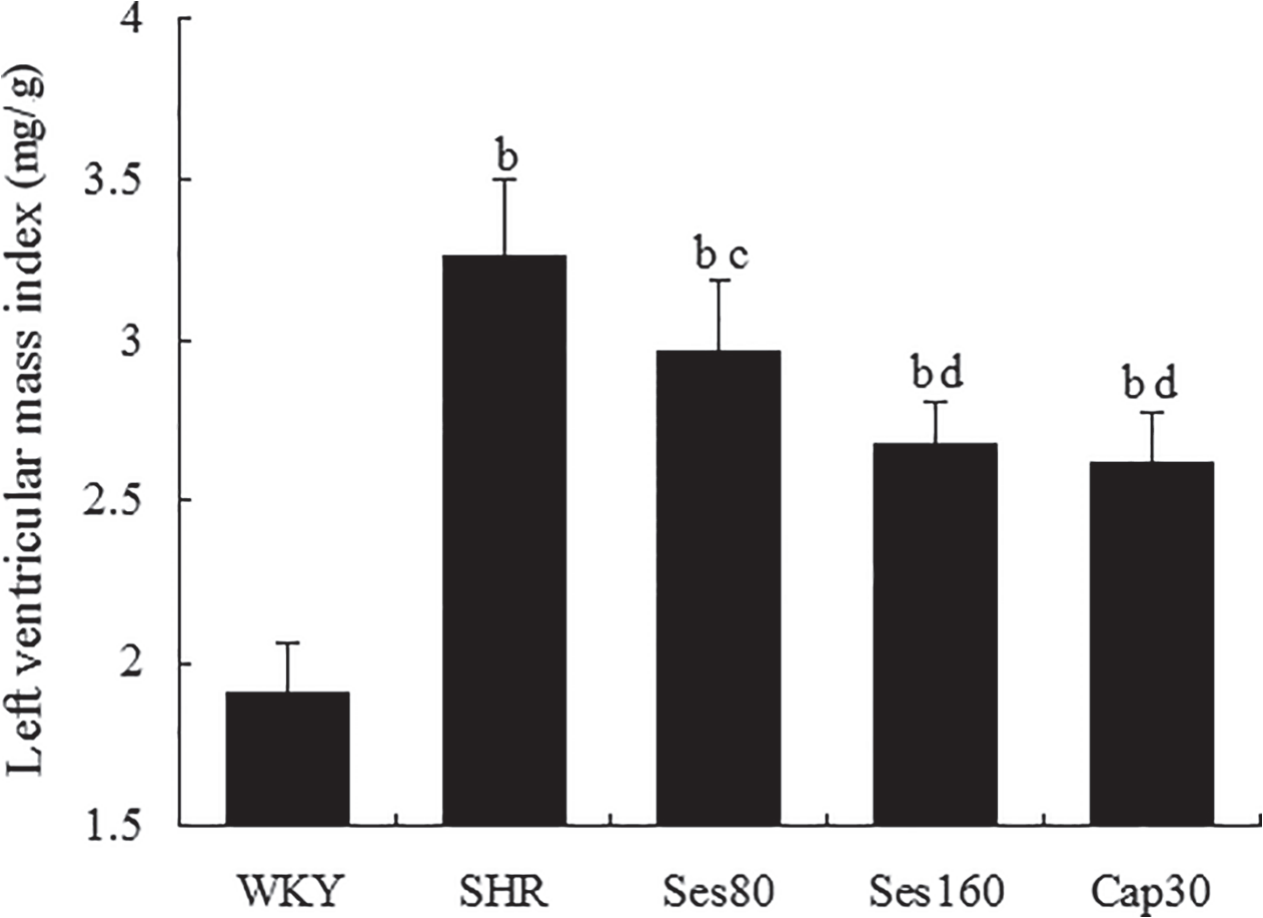
Effect of sesamin on left ventricular mass index (LVMI) in rats. After 12 weeks of supplementation with sesamin or captopril, rats were anaesthetized with pentobarbital sodium and sacrificed, followed by dissection of left ventricles. LVMI is calculated as the ratio of left ventricular weight to body weight (mg/g). Data were presented as mean±SD (n = 7). b *P* <0.01 vs. WKY group; c *P* <0.05, d *P* <0.01 vs. SHR group.

As shown in Fig. 2, LVMI (mg/g) of SHR group was notably higher than that of WKY group (3.28 ± 0.17 vs 1.90 ± 0.12, *P* <0.01), indicating that SHRs developed significant left ventricular hypertrophy. Supplementation with sesamin resulted in a marked reduction in LVMI (3.08 ± 0.14 and 2.90 ± 0.10, *P* <0.05 or *P* <0.01), suggesting that sesamin can effectively ameliorate left ventricular hypertrophy in hypertensive rats. Treatment with captopril also led to a significant alleviation of left ventricular hypertrophy (2.78 ± 0.14, *P* <0.01). Both captopril and sesamin can induce only partial reduction of LVMI in SHRs as compared to WKY group (*P* <0.01).

### Effect of sesamin on left ventricular collagen deposition

**Fig 3:**
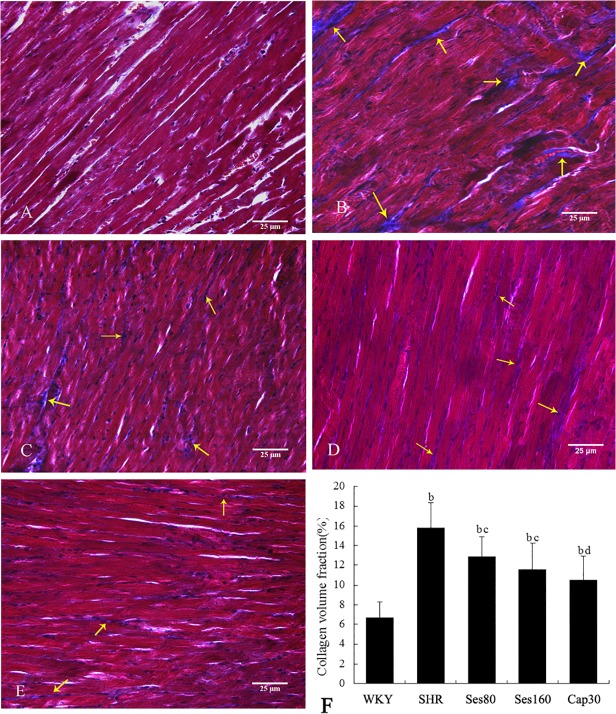
Effect of sesamin on left ventricular collagen deposition in rats. Paraffin sections were subjected to Masson staining, followed by evaluation of collagen deposition with collagen volume fraction (CVF), the mean percentage of collagen area to the total area of each microscopic field. The CVF of each animal represents the mean of 6 randomly selected microscopic fields (400×). Data were presented as mean±SD (n = 7). A: WKY; B: SHR; C: Ses80; D: Ses160; E: Cap30; F: Quantitative analysis of collagen deposition (CVF). b *P* <0.01 vs. WKY group; c *P* <0.05, d *P* <0.01 vs. SHR group.

As shown in Fig. 3, little collagen deposition, which was stained blue by Masson staining, was found in left ventricles of WKY rats (A). By contrast, a large amount of collagen deposition was observed in left ventricular interstitium of SHRs (B), while in sesamin (C, D) and captopril (E) supplemented groups, collagen deposition was reduced obviously. Quantitative analysis with collagen volume fraction (F) indicated that more collagen deposited in left ventricles of SHRs as compared to that in WKY rats (16.14% ± 2.44% vs 6.48% ± 1.57%, *P* <0.01). Supplementation with sesamin (12.87% ± 1.91% and 11.45% ± 2.43%, *P* <0.05) and captopril (10.28% ± 2.25%, *P* <0.01) evidently reduced left ventricular collagen deposition, which remained higher than that in WKY group (*P* <0.01).

### Effect of sesamin on left ventricular contents of type I and type III collagen

**Fig 4:**
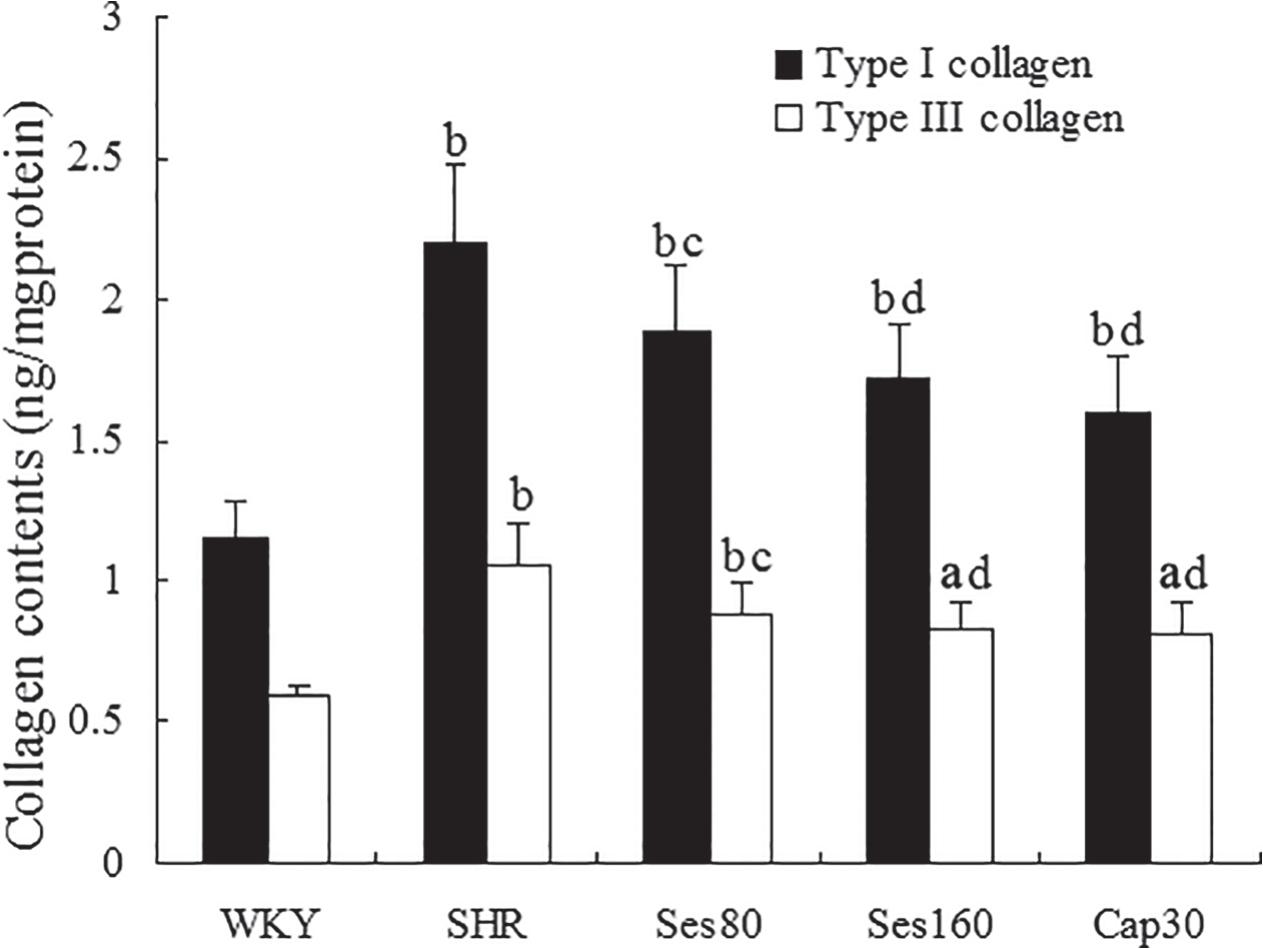
Effect of sesamin on left ventricular contents of type I and type III collagen. Homogenates of left ventricular tissues were determined for contents of type I and type III collagen by ELISA using commercially available kits. Results were normalized to protein concentration determined by bicinochoninic acid assay and presented as mean±SD (n = 7). a *P* <0.05, b *P* <0.01 vs. WKY group; c *P* <0.05, d *P* <0.01 vs. SHR group.

To provide more precise evidence for sesamin’s effect on myocardial fibrosis, we measured left ventricular contents of type I and type III collagen, the two main types of collagen accumulated in hypertensive myocardial fibrosis. As shown in Fig. 4, levels of type I and type III collagen (ng/mg protein) in SHRs were significantly higher than those in WKY rats (2.20 ± 0.28 vs 1.15 ± 0.13 for type I collagen, 1.06 ± 0.14 vs 0.59 ± 0.04 for type III collagen, *P* <0.01), while supplementation with low dose (1.88 ± 0.24 for type I collagen, 0.90 ± 0.11 for type III collagen) and high dose (1.72 ± 0.19 for type I collagen, 0.85 ± 0.10 for type III collagen) of sesamin resulted in a notable reduction in both types of collagen (*P* <0.05 or *P* <0.01). Treatment with captopril also led to a marked reduction of collagen deposition (1.59 ± 0.21 for type I collagen, 0.81 ± 0.12 for type III collagen, *P* <0.01). Left ventricular levels of both type I and type III collagen in sesamin and captopril supplemented groups were still significantly higher than those in WKY group (*P* <0.01).

### Effect of sesamin on plasma and myocardial levels of Ang II

As shown in Table 1, both plasma and cardiac tissue contents of Ang II in SHR group were markedly higher than those in WKY group (*P* <0.01), indicating that significant activation of both systemic and local RAS occurred in SHRs. Supplementation with captopril resulted in a partial but notable reduction in both plasma and cardiac tissue contents of Ang II in SHRs as compared to those in WKY group (*P* <0.05), while sesamin had no significant influence on Ang II contents (*P* >0.05), suggesting that the improvement of hypertensive myocardial fibrosis by sesamin was not derived from inhibition of Ang II production as captopril did.

**Table 1:** Effect of sesamin on plasma and cardiac tissue levels of Ang II (mean±SD, n = 7).

Groups	Plasma Ang II (pg /mL)	Cardiac Ang II (pg/mgprot)
WKY	168. 2±24.2	80.6±13.4
SHR	291.6±32.9 ^b^	144.8±16.5 ^b^
Ses80	278.1±20.4 ^b^	140.0±13.9 ^b^
Ses160	274.7±28.0 ^b^	134.4±25.3 ^b^
Cap30	237.5±23.7 ^b^ ^c^	118.9±12.8 ^b^ ^c^

b *P* <0.01 vs. WKY group

c *P* <0.05 vs. SHR group

### Effect of sesamin on left ventricular levels of TGF-β_1_ protein

**Fig 5:**
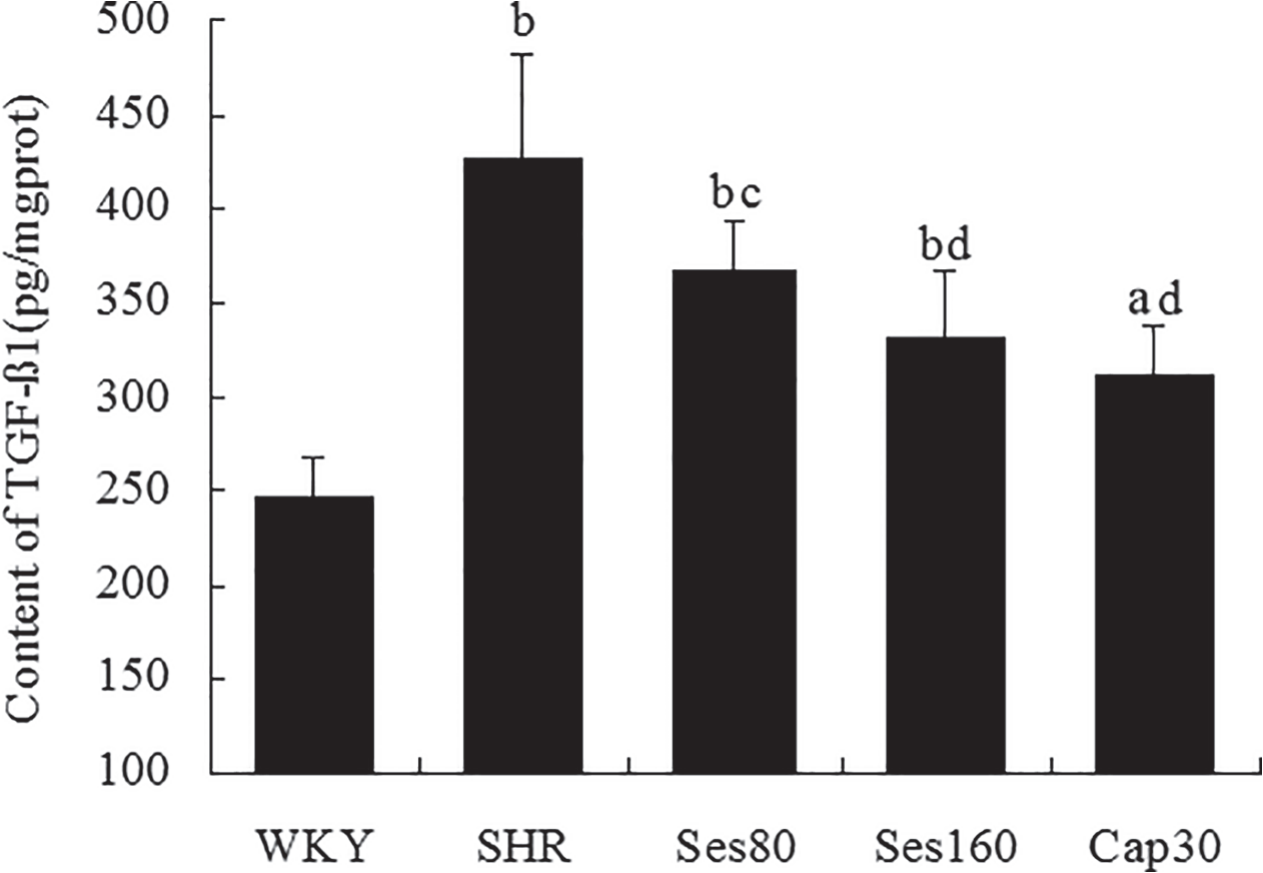
Effect of sesamin on left ventricular levels of TGF-β_1_ protein. After supplementation with sesamin or captopril for 12 weeks, left ventricular homogenates were analyzed for TGF-β1 protein by ELISA. Data were presented as mean±SD (n = 7). a *P* <0.05, b *P* <0.01 vs. WKY group; c *P* <0.05, d *P* <0.01 vs. SHR group.

As shown in Fig. 5, left ventricular level of TGF-β_1_ protein in SHR group was significantly higher than that in WKY group (425.80 ± 55.13 vs 246.26 ± 20.52, *P* <0.01), while in sesamin supplemented groups, levels of TGF-β_1_ protein were decreased evidently (367.01 ± 26.21, 331.54 ± 34.48, *P* <0.05 or *P* <0.01). However, treatment with both doses of sesamin could only promote a partial reduction of TGF-β_1_ protein, which remained significantly higher than that in WKY group (*P* <0.01). Supplementation with captopril also resulted in a marked reduction in TGF-β_1_ protein level (311.63 ± 26.00, *P* <0.01).

### Effect of sesamin on Smad3 phosphorylation and Smad7 protein expression

**Fig 6:**
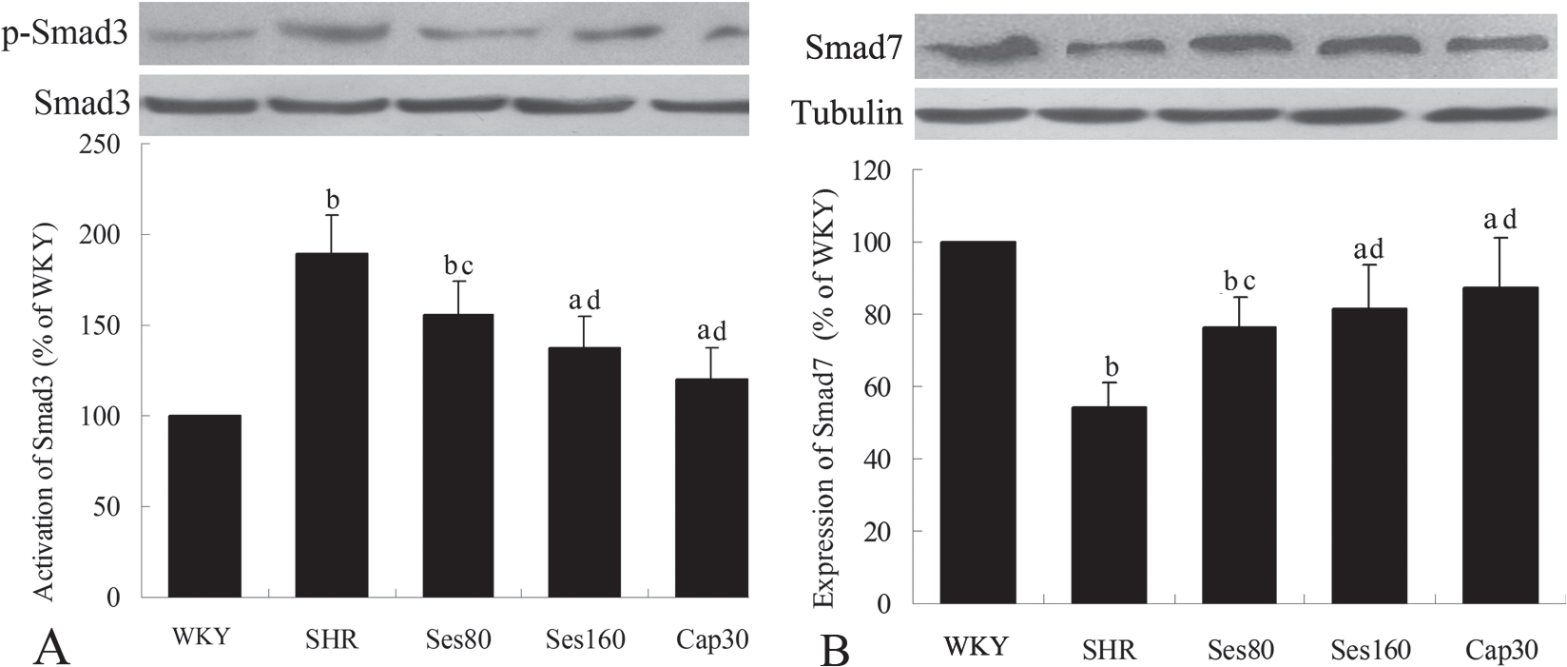
Effect of sesamin on Smad3 phosphorylation and Smad7 protein expression. After supplementation with sesamin or captopril for 12 weeks, total protein were extracted from left ventricular tissues and subjected to western blot analysis as detailed in the Methods section. Levels of p-Smad3 (A) and Smad7 (B) protein were normalized to that of total Smad3 and tubulin, respectively. Data were presented as percentage of that in WKY rats (mean±SD, n = 4). a *P* <0.05, b *P* <0.01 vs. WKY group; c *P* <0.05, d *P* <0.01 vs. SHR group.

As shown in Fig. 6, level of p-Smad3 (A) in SHR group was markedly higher than that in WKY group (189.1% ± 11.3% vs 100%, *P* <0.01), indicating that significant activation of TGF-β_1_/Smad signaling pathway occurred in SHRs. Consistently, protein expression of Smad7 (B), an inhibitor of Smad3 phosphorylation, was notably lower in SHR group than in WKY rats (56.2% ± 5.1% vs 100%, *P* <0.01). Both doses of sesamin significantly suppressed Smad3 phosphorylation (155.3% ± 12.6% and 137.9 ± 11.8%, *P* <0.05 or *P* <0.01), with Smad7 protein expression up-regulated markedly (76.0% ± 5.6% and 80.8% ± 7.0%, *P* <0.05 or *P* <0.01). Supplementation with captopril also resulted in an evident reduction of Smad3 phosphorylation (120.0% ± 7.9%, *P* <0.01) and up-regulation of Smad7 protein expression (87.1% ± 8.2%, *P* <0.01). Both sesamin and captopril can only induce a partial reversion of Smad3 phosphorylation and Smad7 expression in SHRs as compared to those in WKY rats (*P* <0.05 or *P* <0.01). These data suggested that the anti-fibrotic effect of sesamin and captopril in SHRs might be derived from suppression of TGF-β_1_/Smad signaling pathway.

### Effect of sesamin on protein expression of SOD and p47phox

**Fig 7:**
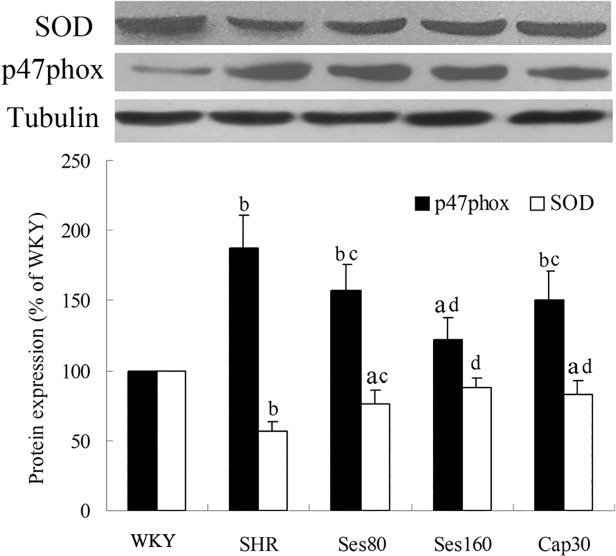
Effect of sesamin on protein expression of SOD and p47phox. After supplementation with sesamin or captopril for 12 weeks, total protein were extracted from left ventricular tissues and subjected to western blot analysis as detailed in the Methods section. Levels of SOD and p47phox protein were normalized to that of tubulin. Data were presented as percentage of that in WKY rats (mean±SD, n = 4). a *P* <0.05, b *P* <0.01 vs. WKY group; c *P* <0.05, d *P* <0.01 vs. SHR group.

As shown in Fig. 7, left ventricular level of SOD protein in SHR group was significantly decreased as compared to that in WKY group (56.8% ± 7.1% vs 100%, *P* <0.01), while the level of p47phox was increased markedly (188.1% ± 15.2% vs 100%, *P* <0.01). Supplementation with sesamin resulted in a significant up-regulation of SOD protein (76.5% ± 9.0%, 88.2% ± 6.3%, *P* <0.05 or *P* <0.01) and down-regulation of p47phox protein (155.0% ± 13.7%, 122.7% ± 13.6%, *P* <0.05 or *P* <0.01). Treatment with captopril also effectively increased SOD protein expression (83.4% ± 7.0%, *P* <0.01) and decreased p47phox protein expression (144.4% ± 16.3%, *P* <0.05). Both captopril and sesamin can only promote partial reversion of SOD and p47phox protein in SHRs as compared to those in WKY rats (*P* <0.05 or *P* <0.01).

### Effect of sesamin on levels of malondialdehyde and total antioxidant capacity

As shown in Table 2, SHRs exhibited a significantly lower level of total antioxidant capacity and higher level of malondialdehyde in left ventricular tissues (*P* <0.01). Supplementation with sesamin and captopril resulted in a notable increase in total antioxidant capacity and decrease in malondialdehyde in left ventricular tissues of SHRs (*P* <0.05 or *P* <0.01). However, both sesamin and captopril can trigger only partial reversion of left ventricular levels of total antioxidant capacity and malondialdehyde in SHRs as compared to those in WKY group (*P* <0.05 or *P* <0.01). These results suggested that sesamin was capable of enhancing antioxidant capacity and alleviate oxidative damage in SHRs.

**Table 2:** Effect of sesamin on left ventricular levels of TAC and MDA in rats (mean±SD, n = 7).

Group	TAC (U/mgprot)	MDA (nmol/mgprot)
WKY	14.1±3.3	14.0±4.5
SHR	5.6±1.9 ^b^	39.3±9.5^b^
Ses80	9.2±3.4 ^b^ ^c^	25.8±8.2 ^b^ ^c^
Ses160	11.3±3.4 ^d^	19.1±4.2 ^a^ ^d^
Cap30	8.6±3.1 ^b^ ^c^	26.4±8.0 ^b^ ^c^

a *P* <0.05

b *P* <0.01 vs. WKY group

c *P* <0.05

d *P* <0.01 vs. SHR group

### Effect of sesamin on Ang II induced secretion of TGF-β_1_ and type I and type III collagen in cardiac fibroblasts

As shown in Table 3, exposure to Ang II for 24 h induced a marked increase in TGF-β_1_ and type I and type III collagen secretion in CFs (*P* <0.01), while preincubation with sesamin of 10.0 and 100.0 μmol/L resulted in a partial but significant decrease in the contents of TGF-β_1_ and type I and type III collagen in culture supernatants (*P* <0.05 or *P* <0.01). Sesamin of 1.0 μmol/L had no significant influence on these measurements mentioned above.

**Table 3:** Effect of sesamin on Ang II induced secretion of TGF-β_1_ and type I and type III collagen in cardiac fibroblasts (mean±SD, n = 6).

Group	TGF-β_1_ (pg/mL)	type I collagen (ng/mL)	type III collagen (ng/mL)
Control	119.38 ± 14.87	2.82 ± 0.34	1.50 ± 0.10
Ang II	183.60 ± 16.25 ^b^	4.56 ± 0.32 ^b^	2.66 ± 0.17 ^b^
Ses (1.0 μmol/L)	169.67 ± 11.74 ^b^	4.17 ± 0.27 ^b^	2.43 ± 0.21 ^b^
Ses (10.0 μmol/L)	161.23 ± 13.62 ^b^ ^c^	3.90 ± 0.31 ^b^ ^d^	2.29 ± 0.24 ^b^ ^c^
Ses (100.0 μmol/L)	148.62 ± 9.19 ^b^ ^d^	3.55 ± 0.22 ^a^ ^d^	1.99 ± 0.20 ^a^ ^d^

a *P* <0.05

b *P* <0.01 vs. Control group

c *P* <0.05

d *P* <0.01 vs. Ang II group

### Effect of sesamin on Ang II induced phosphorylation of Smad3 protein

**Fig 8:**
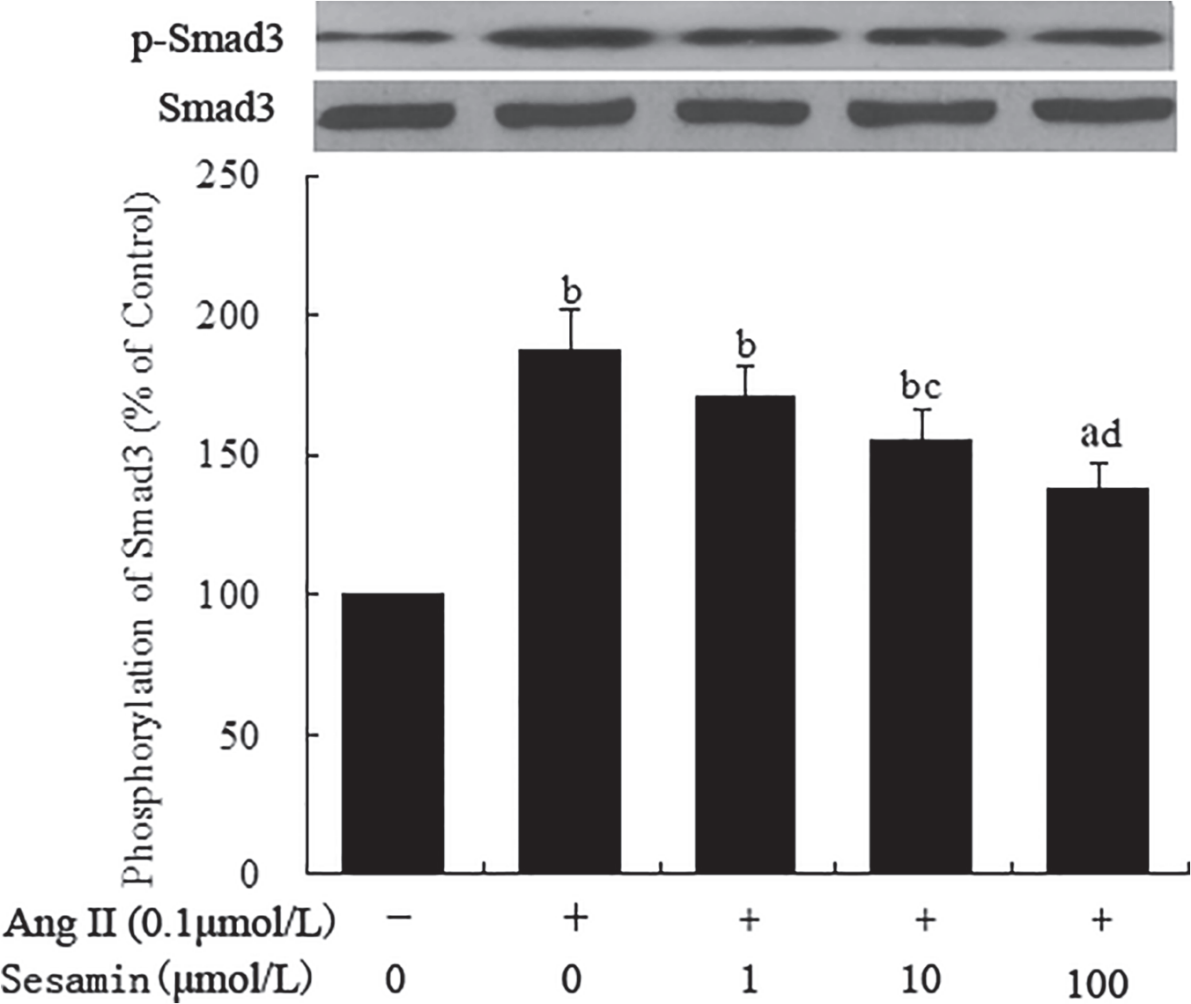
Effect of sesamin on Ang II induced Smad3 phosphorylation. Rat cardiac fibroblasts were isolated from 0 to 3-day-old neonatal rats. Following incubation with sesamin (1.0, 10.0 and 100.0 μmol/L) for 12 h, cells were exposed to Ang II (0.1μmol/L) for 24 h. Cell lysates were subjected to western blot analysis for Smad3 phosphorylation as detailed in the Methods section. Levels of p-Smad3 protein were normalized to that of total Smad3 protein. Data were presented as percentage of that in control group (mean ± SD, n = 3). a *P* <0.05, b *P* <0.01 vs. control group; c *P* <0.05, d *P* <0.01 vs. Ang II group.

As shown in Fig. 8, levels of total Smad3 protein in CFs remained unchanged after exposure to Ang II for 24 h. However, the level of p-Smad3 was notably increased in Ang II treated CFs as compared to that in Control cells (187.9% ± 14.1% vs 100%, *P* <0.01). Preincubation with sesamin of 10.0 and 100.0 μmol/L partially but significantly suppressed Ang II induced phosphorylation of Smad3 protein (155.9% ± 10.6%, 138.1% ± 9.2%, *P* <0.05 or *P* <0.01). Sesamin of 1.0 μmol/L had no significant influence on Smad3 phorsphorylation (170.5% ± 11.3%). These results of in vitro studies indicated that sesamin was able to suppress Ang II induced collagen secretion in cardiac fibroblasts through inhibition of TGF-β_1_/Smad signaling pathway, which was independent of its hypotensive effect.

## Discussion

Increasing evidence indicated that chronic hypertension can induce left ventricular hypertrophy (LVH) through diverse mechanisms [5]. Among the various pathological changes in hypertensive LVH, myocardial fibrosis, a condition characteristic mainly of active proliferation of fibroblasts and over-deposition of extracellular matrix, plays a capital role in left ventricular systolic and diastolic dysfunction and ultimate heart failure [19]. These findings suggest that suppression of myocardial fibrosis might be an effective approach to alleviate hypertensive heart disease. In the present study, SHRs developed significant LVH as manifested by a markedly increased left ventricular mass index (LVMI). Furthermore, Masson staining showed a significant increase of collagen deposition in left ventricles of SHRs. This was evidenced by the markedly elevated levels of type I and type III collagen in left ventricles, suggesting that SHRs developed significant left ventricular fibrosis. By contrast, LVMI and collagen deposition were reduced markedly in sesamin and captopril supplemented groups, suggesting that sesamin and captopril were capable of suppressing left ventricular fibrosis in SHRs.

Previous studies have shown that excessive activation of both systemic and local rennin angiotensin system (RAS) occurred in hypertension, resulting in a significant increase in both circulating and tissue contents of Ang II, a potent pro-fibrotic factor in hypertension [6]. Consistently, the present study demonstrated that both plasma and cardiac tissue levels of Ang II in SHRs were significantly higher than those in WKY rats, while in captopril supplemented group, Ang II contents were reduced markedly. This was consistent with the anti-fibrotic effect of captopril in SHRs, suggesting that the improvement of left ventricular fibrosis by captopril might be related to its suppression of Ang II production. Unlike captopril, however, sesamin did not show significant influence on Ang II levels, suggesting that the anti-fibrotic effect of sesamin is independent of suppression of rennin angiotensin system. As reported previously [20], supplementation with sesamin resulted in a significant lowering of systolic blood pressure in SHRs. Though sesamin was evidently inferior to captopril in reducing blood pressure, however, the improvement of left ventricular fibrosis by sesamin was similar to that by captopril. In vitro studies demonstrated that sesamin was able to suppress Ang II induced secretion of type I and type III collagen in cultured cardiac fibroblasts, in which situation the influence of hemodynamic changes could be precluded. These results suggested that the attenuation of left ventricular fibrosis by sesamin was independent of its blood pressure lowering effect.

To elucidate the possible mechanisms underlying the anti-fibrotic effect of sesamin in SHRs, we examined the impact of sesamin on TGF-β_1_/Smad signaling pathway, a downstream mediator of Ang II induced fibrosis [21]. As a main downstream signal transducer of TGF-β_1_, Smad3 can be phosphorylated by activated type I receptor of TGF-β_1_, followed by forming a complex with smad4 and translocation into nucleus, where it acts as a transcription factor and promotes the expression of target genes including type I and type III collagen. The present study demonstrated that TGF-β_1_ content in cardiac tissues of SHRs was markedly higher than that in WKY rats, and the level of p-Smad3 was also increased markedly. These results suggested that increased Ang II induced significant activation of TGF-β_1_/Smad3 signaling pathway, leading to a marked up-regulation of type I and type III collagen expression. Though sesamin had no evident influence on Ang II contents, however, the levels of TGF-β_1_ and p-Smad3 were reduced markedly in sesamin supplemented groups, accompanied by a significantly reduced levels of type I and type III collagen. Consistent with these in vivo results, in vitro studies also demonstrated that sesamin was capable of suppressing Ang II induced activation of TGF-β_1_/Smad3 signaling pathway in cultured cardiac fibroblasts, indicating that suppression of Ang II induced activation of TGF-β_1_/Smad3 signaling pathway was independent of its blood pressure lowering effect. In addition, sesamin significantly up-regulated the expression of Smad7 protein, which competes with Smad3 for type I receptor of TGF-β_1_ and prevents Smad3 phosphorylation [22]. These results suggested that sesamin could suppress TGF-β_1_/Smad3 signaling pathway, which might contribute to its attenuation of left ventricular fibrosis in SHRs.

It has been proved that hypertension is associated with an increase of reactive oxygen species (ROS) and an impairment of endogenous antioxidant system [23]. During the development of hypertension, increased Ang II can induce oxidative stress not only by activating NADPH oxidase, a predominant source of endogenous ROS, but also by decreasing the activity and protein expression of superoxide dismutase (SOD) [24]. ROS plays a critical role in TGF-β_1_ induced collagen production and differentiation of cardiac fibroblasts into myofibroblasts [24]. The findings that NADPH oxidase inhibitor apocynin and SOD mimetic tempol can effectively reduce Ang II induced collagen production in cardiac fibroblasts provided a potential target for antioxidants in the prevention of hypertensive myocardial fibrosis [25]. Consistently, the present study demonstrated that the expression of p47phox protein, a subunit of NADPH oxidase, was increased significantly in SHRs, while the expression of SOD protein was decreased markedly, suggesting that SHRs developed significant oxidative stress. This was further confirmed by the significantly decreased antioxidant capacity and increased malondialdehyde content in SHRs. Supplementation with sesamin resulted in an evident increase of total antioxidant capacity and protein level of SOD in cardiac tissues, with the levels of p47phox protein and malondialdehyde decreased markedly. These results were consistent with previous reports [14], suggesting that sesamin was able to alleviate oxidative stress in SHRs, which might be one of the potential mechanisms underlying the anti-fibrotic effect of sesamin in SHRs.

In conclusion, the present study demonstrated that sesamin was capable of attenuating left ventricular fibrosis in SHRs through inhibition of TGF-β_1_/Smad signaling pathway, which might be attributed, at least partly, to its enhancement of antioxidant capacity and suppression of ROS production.
